# Victoria, Australia, is getting a new Mental Health and Wellbeing Bill

**DOI:** 10.1007/s11673-022-10212-9

**Published:** 2022-10-03

**Authors:** Chris Maylea

**Affiliations:** La Trobe University, Plenty Rd &, Kingsbury Dr, Bundoora, VIC 3086 Australia

**Keywords:** Mental health, Law, Victoria, Mental Health and Wellbeing Act, Mental Health and Wellbeing Bill

## Introduction

French mathematician and philosopher Blaise Pascal wrote^1^, of a letter, that “I have made this longer than usual because I have not had time to make it shorter”. The drafters of the Victorian *Mental Health and Wellbeing Act 2022* (the *Act*) may make a similar claim, having drafted Australia’s longest ever piece of mental health legislation in the shortest possible time. At 686 pages, the Act surpasses the previous *Mental Health Act 2014* (Vic), which ran to 351 pages, and is five or six times as long as the *Mental Health Act 2007* (NSW) (at 115 pages) or the *Mental Health Act 2009* (SA) (at 95 pages).

Is the Victorian Act five or six times better than its counterparts across the border? Does the considerable heft result in increased rights protections for people detained in mental health services or subject to compulsory mental health treatment in the community? The simplistic answer to this is that no, the Act does little to directly improve the experience of people subject to it. The longer, more nuanced answer is; maybe. The Act contains a range of system level changes, new bodies and provides an underpinning for a new mental health system which may, over many years, result in an improved experience. There are also some symbolic elements that are welcome, if likely to be substantially ineffective. I will first introduce the context for the new legislation, then cover the symbolic elements, before turning to the systemic changes then considering changes to the direct experience. I conclude with a discussion of the next steps in the process.

## Context

It is widely accepted that Victoria’s mental health system has failed to meet the needs or protect the rights of Victorians experiencing mental distress (Royal Commission into Victoria’s Mental Health System 2021). This is only the latest in a long line of damning investigations into mental health services, both in Victoria and across Australia. People subject to mental health legislation consistently and repeatedly report rights violations and inconsistent application of the law (Maylea et al. 2022). Use of force varies wildly, with some services using seclusion at rates fourteen times higher than others (VMIAC 2022). Some services almost never use mechanical restraint, while others regularly restrain people. Victoria has the highest rates of community compulsory treatment in Australia, and some of the highest rates in the world, although easily comparative data is not publicly provided (Light 2019). This use of force is not a “regrettable necessity”, as many similar jurisdictions manage to provide mental health services with much, much less use of force. Some services have nearly eliminated the use of force altogether (Zinkler 2022). People who are subject to these rights violations are often traumatised by the experience and are much less likely to trust or use mental health services in the future.

These systemic failures are particularly acute in light of Victoria’s *Mental Health Act 2014* having been only fairly recently enacted in the wake of the *Convention on the Rights of Persons with Disabilities* (CRPD) (United Nations 2006). The CRPD has the potential to herald a new era of rights-based approaches to mental health and other disability law, policy and practice, abolishing the use of force and coercion, but in most of the world that vision remains unrealised. Some countries have attempted to implement CRPD-compliant mental health legislation, such as Costa Rica, Peru and Colombia (WHO 2021). Peru, for example, abolished guardianship on the basis of disability, and its *2020 Mental Health Law* does not allow treatment without consent. Northern Ireland has laid out a less ambitious plan that should help reduce discrimination on the basis of disability (Harper, Davidson, and McClelland 2016). Elsewhere in the United Kingdom, Europe, the United States, Canada, Australia and New Zealand, the focus has been on reducing the negative impacts of compulsory treatment, rather than abolition. In all these cases, we are yet to see how effective these reforms have been, although furious debate continues.

This furiousness is common in discussions of mental health law reform, where there is no consensus or accepted evidence base for what mental health law should be trying to achieve, or how to measure its success. Is mental health law intended to protect people who experience mental distress, to protect the public from “dangerous mental health patients”, to limit the harms inflicted by compulsory treatment, or to provide cover for an underfunded and poorly structured mental health system that fails to provide support for people who require it? The best available evidence indicates that different approaches to legislation–such as legislation focused on treating people who are “dangerous” or treating people who “lack capacity” to make decisions for themselves–do not seem to result in obvious differences in measurable system level outcomes (Rains et al. 2019). Put simply, we do not know what works and what does not in terms of mental health law reform–even if we could agree on what we are trying to do.

It is widely accepted that the implementation of the Victorian 2014 legislation was unsuccessful, or at least unsuccessful at driving down rates of compulsory treatment and ensuring rights protections. In 2019, the Victorian Government announced a Royal Commission into Victoria’s Mental Health System. In February 2021, it handed down its final report, with recommendation 42 of 65 calling for a new *Mental Health and Wellbeing Act* to be enacted by mid-2022. This gave the team drafting the Act three months to draft proposals for public consultation, incorporate feedback from consultation for approval in October 2021, for cabinet approval by April 2022, for tabling in May. This timeline did not run quite to plan, but, against all odds, the bill was passed in June 2022 and will come into force in 2023. This breakneck pace is unheard of in mental health legislative reform, which ordinarily takes place over years, not months. The pace attracted some criticism from the sector, which appears wary of fundamental change from its current, depleted position. The new legislation is more about providing for urgent improvements to the service system than addressing issues of rights protections and rights violations. It may also be that the impending November 2022 Victorian election influenced this timeline, with the Royal Commission seeking to have its recommendations legislated before the political cycle turned away from mental health reform.

As such, most of the changes in the new legislation relate to Royal Commission recommendations, and in no way attempt to fulfil Australia’s obligations under the CRPD. The criteria for assessing if a person can be apprehended, detained, secluded, restrained, and/or medicated remain the same. People will still receive electroconvulsive treatment against their will. No significant changes have been made to advance planning. Compulsory treatment will continue despite calls for its immediate abolition from the United Nations and the World Health Organisation (Committee on the Rights of Persons with Disabilities 2014; WHO 2019). Partially in response to community critique, the Victorian Government has announced that it will bring forward an independent review which will consider the compulsory treatment criteria, compulsory treatment safeguards, and alignment with other medical decision-making legislation, although not CRPD compliance.

## Symbolic changes

The Act contains some largely symbolic elements that, while welcome, are unlikely to be meaningful by people subject to it. Central to this are changes to the Principles and Objectives, which are now more rights based and reflect more contemporary language. These are welcome, however, as the criteria for deciding if a person can be subject to compulsory treatment remain unchanged, it is unclear to what extent decision-makers will be influenced by these changes in their daily practice. The Principles and Objectives of the existing legislation are not always explicitly referenced in day-to-day decision making, so this change may yet prove to be largely symbolic.

Similarly symbolic, the Act recognises that seclusion and restraint provide no therapeutic benefit and highlights the intention of eliminating seclusion and restraint within ten years but it does not actually legislate for this to occur. The Act continues to provide for the use of seclusion and restraint.

The Act also recognises the Victorian government’s commitment to Aboriginal self-determination, which is a nice touch, but stops short of giving legislative power to the *United Nations Declaration on the Rights of Indigenous Peoples*, which would be a more tangible and impactful move. It is difficult to see anything tangible that will contribute to decolonization, let alone reduce the overrepresentation of First Nations people in the Victorian mental health system.

## Systemic changes

The new Act has a much broader scope than the current legislation, including providers of wellbeing and other mental health services, not just public mental health services. It establishes a new Mental Health and Wellbeing Commission, a collaborative research centre, regional mental health and wellbeing boards, Regional and Statewide multiagency panels, a youth mental health statutory body and replaces the Deputy Secretary, Mental Health with a more powerful Chief Officer for Mental Health and Wellbeing.

The new Mental Health and Wellbeing Commission is largely a rebadge of the existing, largely ineffective Mental Health Complaints Commissioner, with some additional powers to publish data, launch “own motion” investigations and hear complaints from carers and families as well as from people in the system. Its success will depend on the extent it is willing to aggressively police rights violations in the mental health system and to use its powers to hold mental health services to account.

These systemic changes, alongside significant investment paid for by the newly legislated mental health levy, have the potential to revolutionise Victoria’s mental health system. The success of these reforms will not be clear for years, if not decades. Certainly, very few of the people taken to hospital against their will, detained, secluded, restrained and/or forced to receive treatment in the community, will notice any immediate improvement in their treatment, care and support as a result of these systemic changes. Perhaps, over time, coupled with other changes, we might see some improvement in rights protections, reduced use of force and coercion and subsequent trauma.

## Changes to the experience of people made subject to the new legislation

What, then, will a person subject to the new legislation notice? There are two main things that will hopefully be immediately clear. The first is that now, rather than police being the primary responders to people experiencing mental distress in the community, there will be a “health-led response”. The second is that all people who are subject to compulsory treatment will be offered an independent, non-legal mental health advocate.

The health-led response is based on Royal Commission recommendation 10, which seeks to ensure that health professionals, rather than police, respond to people in mental health crisis in the community. Where police are also in attendance, health professionals should lead the response. This is a welcome change, but not a particularly substantial change, as these health professionals will be paramedics, not mental health professionals or peer workers. People do prefer paramedics to police, but this presents a missed opportunity to implement genuine alternatives which are not focused primarily on transport to emergency departments. The legislation simply gives police-like powers to paramedics, who will now be able to detain people in the community, or rather, in the parlance of the Act, take people into their “care and control”. Paramedics could already enter a person’s private premises, search, seize and secure items and use bodily restraint to take a person to a mental health service, so this change extends these powers into the community. This is hardly a major human rights victory, even if it is likely to improve the experience of some people while they are detained.

The other change that people are likely to notice is opt-out advocacy. Advocacy is currently provided by Independent Mental Health Advocacy, but many people are not aware they are able to access an advocate. Under the new legislation, consistent with Royal Commission recommendation 56, psychiatrists will be required to notify the advocacy service that a person is subject to involuntary treatment. This is another welcome change, as advocacy has been shown to improve rights protections, support decision-making and provides an avenue of access for people seeking lawyers for legal representation. As with the “health-led” response, this is not a substantial change, but a welcome one.

## Discussion

There is a grab bag of other changes in the legislation, such as changes to information sharing, that are unlikely to result in significant change in practice but represent tidier drafting. The takeaway is that the legislation is not even attempting to bring about direct reform to practice; rather it is attempting to provide the foundations for the system that will eventually bring about the intended reform. The then Minister for Mental Health, James Merlino, said as much in the second reading speech:

*It would be naive to expect that the reforms of the royal commission can be implemented overnight. It took us so many years of underinvestment to get the broken system described by the royal commission, and it will take at least a decade of unwavering commitment to this reform to build the system that Victorians deserve. Legislation alone cannot mend a broken mental health system, and this bill will not—and cannot—be all things to all people. But my sincere hope is that it represents a significant leap forward in the legal foundations of this work, building new system leadership, establishing a wellbeing and rights-based approach to mental health and centring voices of lived experience.*

What is missing in this “sincere hope” are the real changes required to reorient Victoria into CRPD compliance. This would mean following Peru’s move to abolish compulsory treatment and guardianship based on disability, replacing the current mental health legislation with a system that provided support required for people to make their own decisions (Committee on the Rights of Persons with Disabilities 2014). Short of full CRPD compliance, the legislation is not even compliant with the local Victorian *Charter of Human Rights and Responsibilities Act 2006*, as it discriminates against people based on their diagnosis. Changes to comply with local human rights legislation would not be too difficult, as is demonstrated by the provision of binding advance directives in the Australian Capital Territory *Mental Health Act 2015* or the prohibition of treating people who have decision-making capacity under the Western Australian *Mental Health Act 2014*. Victoria will continue to deny people in the mental health system the same rights as those held by people in the physical healthcare system; the right to advance planning and the right to refuse treatment when capacious. This is also acknowledged by the Victorian government, in the statement of compatibility with human rights that must accompany all new Victorian legislation:

*The compulsory treatment provisions may potentially amount to direct discrimination on the basis of disability. Direct discrimination occurs where a person treats a person with an attribute unfavourably because of that attribute. The provisions treat people with mental illness differently from other people on the basis of their mental illness. The provisions also treat people with a mental illness differently from people with a physical illness because the Bill allows treatment without consent in circumstances where the* Medical Treatment, Planning and Decisions Act 2016 *does not—namely where a person has capacity.*

Despite this acknowledgement, the Victorian government cheerfully claims that the Act is compatible with local human rights legislation. As there is no mechanism for judicial review of these claims, they will stand untested.

That the Victorian government has already announced a legislative review that will begin as soon as the legislation is a clear indicator that the legislative reform process is not actually finished. This review will consider the treatment criteria, compulsory treatment, and alignment with other medical decision-making legislation, but not CRPD compliance. It is possible that this review may result in recommendations that do result in real rights protections and something resembling real compliance with the *Charter of Human Rights and Responsibilities Act 2006* (Vic), although the limited scope of the review means that it will not be able to consider the full range of issues that would be required to achieve CRPD compliance.

In any case, we are some years away from seeing what the recommendations of this review will be and if they will be implemented by the government of the day. We are many years, perhaps decades, from seeing what the impact of the broader systemic reforms will be. It will be impossible, at that point, to highlight any specific element that contributed to the failure or success of the reforms, let alone the role played by legislation in this very complex ecosystem. Hopefully, at some not-too-distant point in the future, we will be able to look back and reflect on the success of these reforms in the elimination of seclusion and restraint, the elimination of force and coercion (or more likely a substantial reduction of the use of force and coercion) and a mental health system that is genuinely appreciated by the people it serves. More likely, I suspect, the failure to take this opportunity to ensure a fully human rights compliant mental health system will see us back in this same situation with the next damning report, and, likely as not, more damning reports after that.

## Footnotes

1 Blaise Pascal (1623-1662) in *Lettres provinciales* (Lettre XVI)
